# Reply to Comment On: “Indirect Assessment of Skeletal Muscle Glycogen Content in Professional Soccer Players before and after a Match through a Non-Invasive Ultrasound Technology *Nutrients* 2020, *12*(4), 971”

**DOI:** 10.3390/nu12072066

**Published:** 2020-07-12

**Authors:** Iñigo San-Millán, John C. Hill, Julio Calleja-González

**Affiliations:** 1 Department of Medicine, Division of Endocrinology, Metabolism and Diabetes, University of Colorado School of Medicine, Aurora, CO 80045, USA; INIGO.SANMILLAN@cuanschutz.edu; 2 Department of Human Physiology and Nutrition, University of Colorado, Colorado Springs, CO 80918, USA; 3 Department of Family Medicine, University of Colorado School of Medicine, Aurora, CO 80045, USA; john.hill@cuanschutz.edu; 4 Laboratory of Analysis of Human Performance, Department of Physical Education and Sport, Faculty of Education, Sports Section, University of the Basque Country, 01007 Vitoria, Spain

We would like to thank Professor Niels Ørtenblad et al. for carefully reading and commenting [1], on our recent publication. In our paper published in this journal, we present a pilot study application of a novel way to “indirectly assess” skeletal muscle glycogen based on the methodology that we developed though high-frequency skeletal muscle ultrasound [2]. The present published work is a pilot study that may offer considerable applications in the field of team sports. In general, our methodology is based on the correlation between skeletal muscle echogenicity (sound intensity) and the glycogen content from skeletal muscle biopsy. In our validation study, these correlations were quite robust both pre-exercise (r = 0.94, *p* < 0.001) and post-exercise (r = 0.93, *p* < 0.001) [2].

Our methodology does not simply “*convert pixilation intensities in ultrasound images to a score of glycogen content based on muscle water content*”, as the authors state. The science behind skeletal muscle ultrasound is more complex than that. Ultrasound is a well-documented imaging modality that objectively measures the densities of tissues based upon their water content. As with all imaging modalities, such as radiography or positron emission tomography (PET) scans, it is an indirect measure. For example, the 18F-Fludeoxyglucose F 18 injection (FDG)-PET scan is a widely used imaging technique for cancer diagnosis. Fludeoxyglucose F 18 injection (FDG) is a positron-emitting radiopharmaceutical containing radioactive 2-deoxy-2-[^18^F] fluoro-d-g1ucose, which is used in conjunction with position emission tomography (PET) for cancer diagnosis. Due to the Warburg effect, cancer cells utilize an exacerbated amount of glucose and produce a lactate, probably for lactagenesis purposes [3]. However, this technique does not directly detect cancer, as 18F-FDG-PET simply exposes those tissues with an exacerbated glucose uptake, which could be a surrogate for the Warburg effect. In fact, the use of 18F-FDG-PET in brain tumors may have some diagnostic limitations due to the physiologically high glycolytic activity of brain tissue. Furthermore, it is well accepted that X-rays do not actually represent bone, but are images of bone. The same is true for ultrasound. Echogenicity is the key concept behind the science of skeletal muscle ultrasound. Skeletal muscle echogenicity is not just “water content”, as water is only one of the different echogenic components of skeletal muscle ultrasound. We are surprised that the word “echogenicity” is not mentioned by the authors, either in their letter or in their studies. Since any study describing how ultrasound works should allude to the echogenicity status of the muscle (i.e., hyperechoic vs. hypoechoic), we believe that the authors misinterpret the science of ultrasound technology. 

Moreover, the authors claim that there are two validation studies disproving our methodology (Routledge et al. and Bone et al.). However, only one of these studies has been published in a peer-reviewed journal [4]. The other study by Bone and colleagues [5] was a poster presentation at the American College of Sports Medicine ACSM annual meeting in 2016 and was never published in a peer-reviewed journal. Furthermore, both studies introduced variables that interfered with the water balance of the muscles. 

To clarify, our validation study correlated the glycogen content from an ultrasound-guided muscle biopsy obtained from the rectus femoris muscle with the echogenicity from the exact image obtained where the muscle biopsy was performed (Figure 1). On the other hand, both Morton and Burke’s groups utilized a different approach and methodology, correlating glycogen content from the muscle biopsy with the echogenicity of the entire muscle. These studies were conducted with the traditional blind muscle biopsy technique of the vastus lateralis muscle instead of the precisely targeted ultrasound-guided biopsy technique we used.

Furthermore, Routledge and colleagues performed the post-exercise biopsy “*within 40 min upon completion of match play*”. These muscle biopsies were obtained during the recovery period and were not obtained immediately after the game, which can result in artifacts. The first and probably most important artifact is related to the eccentric loading of muscles. This can produce microtrauma, which will cause the tissues to become edematous (hypoechoic). Waiting more than half an hour to perform an ultrasound post-game introduces a confounding variable. The study design does not recognize this imaging limitation and assumes that all water changes in the tissue are due to shifts in glycogen. In addition to the microtrauma, glycogen re-synthesis post-exercise, even in the absence of carbohydrates (CHO) intake, will take place. It is estimated that glycogen synthesis from gluconeogenesis occurs at rates of 1–2 mmol·kg wet wt of muscle^−1^·h^−1^ [6,7]. Lactate can also be an important gluconeogenic precursor and can be converted to glycogen [8]. In fact, in a study by Hermansen and colleagues, muscle glycogen content was rapidly increased (+32%) without CHO intake in all of subjects 30 min post maximal exercise. This is especially important in the case of high-intensity exercises, like in the case of the study conducted by Routledge and colleagues, as there is an important production of lactate during team sports. Routledge and colleagues performed muscle biopsies within 40 min after the game; therefore, it is very possible that, by that time, all athletes could have significantly increased their glycogen stores, as reported by Hermansen and colleagues.

Moreover, Fernandez-Elias and colleagues showed that, after prolonged exercise, muscle water content was significantly higher in those athletes who fully replaced the water lost during exercise vs. those who had a low fluid intake post-exercise [9]. In this study, muscle glycogen content was replenished equally among the two groups, independent of muscle water content. However, during the recovery phase (1 h post exercise) the fully rehydrated group had a ratio of 17 g of water per gram of glycogen (17:1) compared to 3:1 in the group that did not fully rehydrate. The significant increase in higher water content 1 h post exercise due to full rehydration would represent an artifact for the echogenicity of the ultrasound image. The supra-physiological amount of water in the muscle will produce hypoechogenicity in athletes within 1 h post-exercise. Ultrasound measurements should be performed immediately post-exercise to control for this confounding variable.

Routledge and colleagues did not take into consideration the amount of water consumed by their subjects within 40 min of recovery post-exercise. The total amount of muscle water related to water consumption could add to this artifact. Performing delayed muscle biopsies and skeletal muscle ultrasound assessments post-exercise will pose serious confounders. If the limitations of ultrasound imaging are not recognized or acknowledged, then it is possible to design a study where confounding variables will negate the relationship of water to glycogen. Therefore, we believe that the data obtained in this study are not reliable due to multiple confounders.

The authors claim that there is little variation in the glycogen between different sites of skeletal muscle, citing Harris et al.’s article from 1974. In a study published in 2013, Dr. Ørtenblad states that “*The use of electron microscopy has revealed that glycogen is not homogeneously distributed in skeletal muscle fibres, but rather localized in distinct pools*” [10]. Furthermore, Ørtenblad and colleagues state in the aforementioned article that “*each glycogen granule has its own metabolic machinery with glycolytic enzymes and regulating proteins*”. The main knowledge about muscle glycogen concentration is derived from mixed muscle tissue and it is well known that skeletal muscle is composed of fast- and slow-twitch muscle fibers with different rates of contraction and metabolic properties. Type I and type II muscle fibers are recruited differently during exercise in response to the metabolic demands of exercise. For example, pre-exercise muscle content is higher in type IIa muscle fibers than in type I muscle fibers [11]. However, glycogen content is lower in type I muscle fibers than in type II post-running [12,13], but lower in type II fibers compared to type 1 post-jumping [12]. Furthermore, it is not clear whether different contraction patterns, glycogenolysis and gluconeogenesis rates during exercise would be identical and homogeneous throughout different portions of the muscle (e.g., superficial vs. deep muscle; distal vs. proximal portion of the muscle). Since a given skeletal muscle contains different muscle fibers, which have distinct glycogen storage pools, different metabolic activities and also different muscle fibers, recruitment patterns and metabolic characteristics, it is plausible to believe that glycogen cannot be stored uniformly throughout that given muscle and that a single muscle biopsy can neither represent the glycogen content of the entire muscle nor the metabolic and glycogenolysis rates across that given muscle. We believe that this is an assumption that has endured for decades. For example, the pioneer study done by Hermansen and colleagues in 1967 showed that glycogen from muscle biopsies in ten trained subjects was almost entirely depleted (1.6 to 0.06 g⋅100 wet muscle) after 90 min at an average exercise intensity of 77% of VO2max and a CHO oxidation rate of 2.8 g·min^−1^ [14]. Over half a century later, there are some doubts about these earlier studies showing almost complete glycogen depletion after 90 min of moderate exercise. Many people exercise for 90 min at an intensity of around 77% of their VO2max and do not “hit the wall” or “bonk”. In fact, multiple nutritional guidelines do not even recommend CHO supplementation for efforts lasting less than 90 min. 

The fact that a simple muscle biopsy probably does not represent total glycogen content from a given muscle is the main reason for the MuscleSound methodology, which is based on the correlation between the glycogen content of a specific muscle biopsy and the echogenicity of that specific muscle, presenting an attractive, non-invasive and novel methodology to indirectly assess glycogen content in muscles. 

Regarding the glycogen depletion rate, the authors state that they are struck by the fact that the average glycogen reduction was only 20% compared to one muscle biopsy study showing a 50% reduction in soccer players [15]. There are several studies assessing glycogen content from a muscle biopsy in soccer. It is worth noting that there are significant differences among studies. For example, Saltin reported that at the end of a friendly soccer game, the glycogen content from a muscle biopsy was almost gone (91% decrease) [16]. In a different study during a competitive soccer game, Currie and colleagues showed a 31% decrease in glycogen between pre- and post-game muscle biopsies [17]. In a semi-competitive game, Leatt et al. showed a 32% reduction in glycogen between pre- and post-game muscle biopsies [18]. Neither Saltin nor Currie and colleagues reported precise CHO intake during exercise. However, Leatt and colleagues reported an administration of 35 g of CHO 10 min before the game and another 35 g of CHO at half time (70 g total), resulting in a 32% decrease in glycogen content at the end of the game. In our study, we reported a total intake of 105 g of CHO before the game and at half time (~35% higher total CHO than Leatt and colleagues), which is in the range or higher than the 30–60 g·h^−1^ recommended for soccer players [19]. Therefore, our results are not dissimilar to what Leatt and colleagues reported. Furthermore, De Bock and colleagues administered 1 g CHO·kg body weight^−1^·h^−1^ to subjects performing exercise at ~75% of VO2max for 120 min [11]. They showed that the glycogen content in Type II muscle fibers was reduced by 19% in the CHO group compared to ~53% in the placebo group without CHO. Moreover, referring back to the pioneer study by Hermansen and colleagues from 1967, they observed almost a complete depletion of glycogen from a muscle biopsy without CHO intake after 90 min at an intensity of 77% of VO2max or a glycolytic intensity of 2.8 g·min^−1^ of CHO oxidation [14]. The protocol for our validation study was almost identical, as subjects pedaled for 90 min at an intensity eliciting 2–3 g·min^−1^ of CHO oxidation and between 70–80% of VO2max [2]. However, we observed significantly different results with a decrease in glycogen content from a muscle biopsy of ~36%, which is also in agreement with the different studies we mentioned [17,18] in soccer, a sport whose average intensity is ~70% of VO2max [15] with high glycolytic bursts. 

Lastly, the authors state that we misinterpreted the results of a study by Ørtenblad and colleagues [20]. From the results of this study, we interpreted that a ~25% decrease in muscle glycogen corresponds to a ~10% decrease in SR Ca^2+^ release and uptake. The authors add that this association only seems to occur when glycogen levels are reduced below ~50% of resting levels. We appreciate this clarification, as it helps us to understand better this particular finding. Nevertheless, the intention of our study was not to delve into the relationships between muscle glycogen content and SR Ca^2+^ release and uptake. We simply intended to praise the authors’ novel approach to associate muscle glycogen content with SR Ca^2+^ release and uptake, which, as we state in our article, “*could have important consequences for athletic performance*”. With that being said, the findings in this study [20] showed weak correlations between transmission electron microscope-determined subtractions of glycogen and SR Ca^2+^ release rate for intermyofibrillar glycogen (r^2^ = 0.08, *p* = 0.23), intramyofibrillar glycogen (r^2^ = 0.23, *p* = 0.04) and subsarcolemmal glycogen (r^2^ = 0.12, *p* = 0.14), as well as with SR Ca^2+^ release rate and total glycogen concentration from a biopsy (r^2^ = 0.29, *p* < 0.001). Nevertheless, we believe that this was a novel approach to try to investigate possible mechanisms by which muscle glycogen content could play a role in fatigue, muscle contraction and athletic performance. 

## Figures and Tables

**Figure 1:**
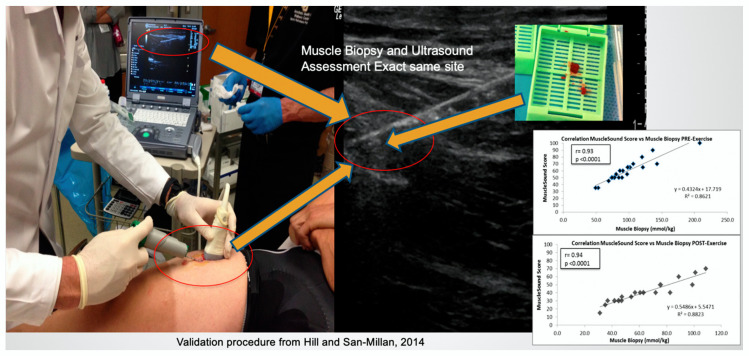
Validation procedure through ultrasound-guided muscle biopsy (Hill and San-Millan, 2014). The ultrasound-guided muscle biopsy allows to determine the exact site from where the muscle biopsy was obtained. Only through this procedure is possible to correlate the glycogen content from the muscle biopsy with the echogenicity of the image corresponding to the muscle biopsy site.
